# Supplementary material for: Impact of distinct FG nucleoporin repeats on Nup98 self-association
Source: Nat Commun. 2024 May 7;15:3797. doi: 10.1038/s41467-024-48194-4 (PMC11076500; doi:10.1038/s41467-024-48194-4)
Supplement: Supplementary file 1 — Supplementary Information [file 41467_2024_48194_MOESM1_ESM.pdf]

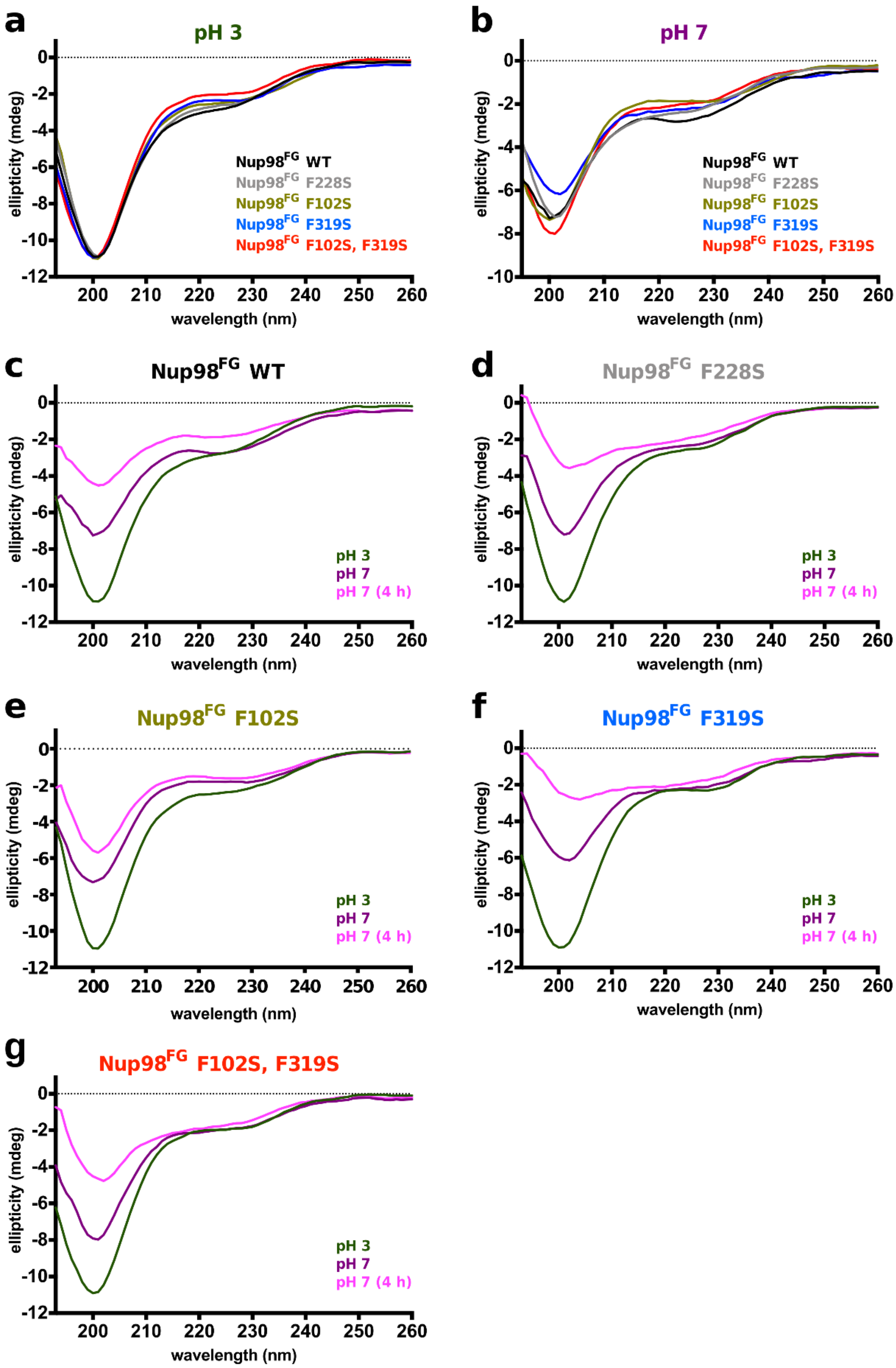

Supplementary Figure 1 | CD spectra of Nup98^{FG} mutants. a-b, CD spectra of the different Nup98^{FG} mutants (WT, black; F228S, grey; F102S, dark yellow; F319S, blue and F319S+F102S, red) at pH 3 (a) and 7 (b). **c-g,** CD spectra of Nup98^{FG} WT (c), F228S (d), F102S (e), F319S (f) and F102S+F319S (g) at pH 3 (dark green), pH 7 (dark purple) and pH 7 after 4 hours (pink). Source data are provided as a Source Data file.

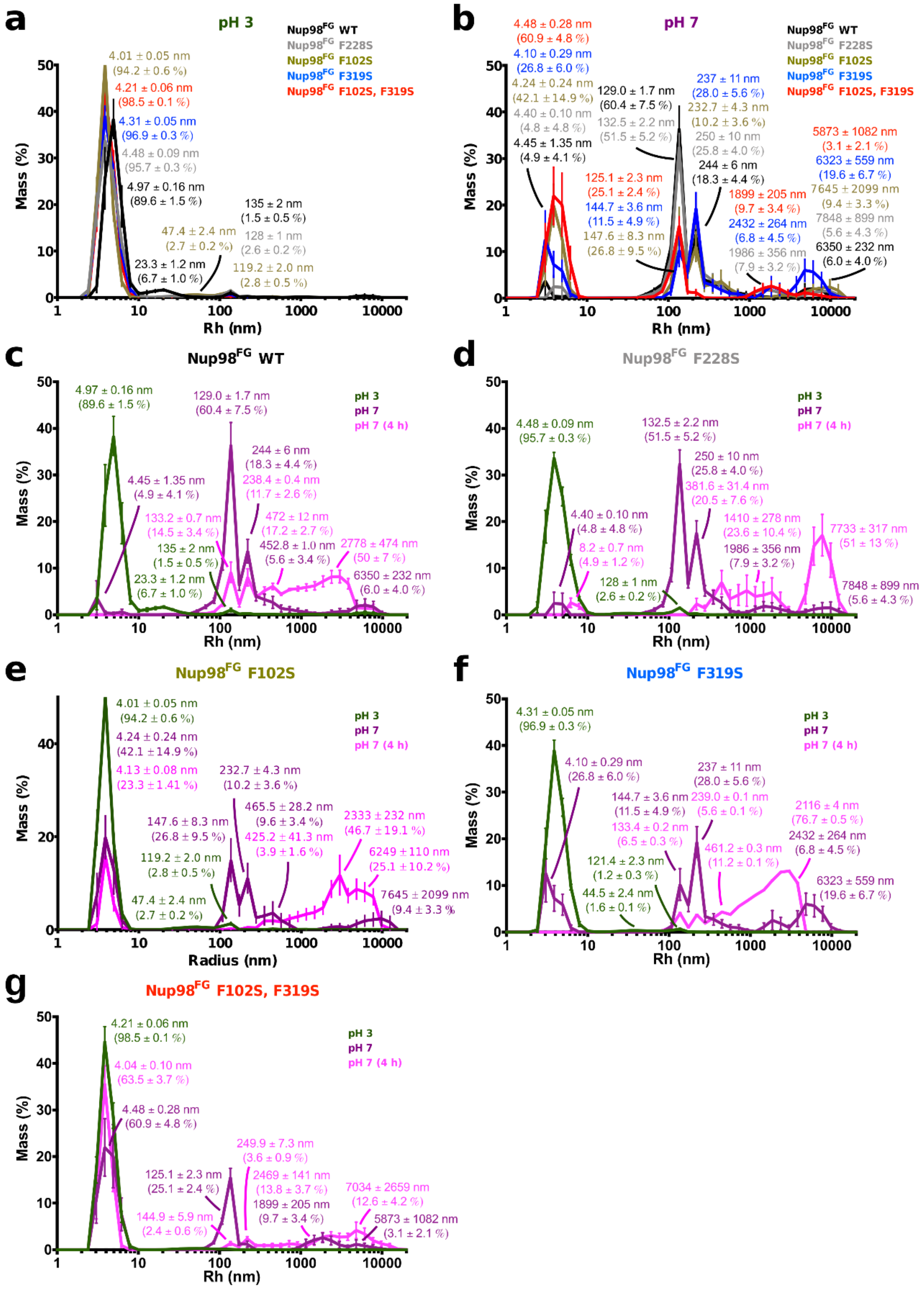

Supplementary Figure 2 | Hydrodynamic radii of Nup98^{FG} mutants. a-b, Hydrodynamic radii of the different Nup98^{FG} mutants (WT, black; F228S, grey; F102S, dark yellow; F319S, blue and F319S+F102S, red) at pH 3 (a) and 7 (b) measured by DLS. **c-g,** Hydrodynamic radii of Nup98^{FG} WT (c), F228S (d), F102S (e), F319S (f) F102S+F319S (g) at pH 3 (dark green), pH 7 (dark purple) and pH 7 after 4 hours (pink). The sizes, together with the population percentages in mass and the respective standard errors are displayed next to each peak (n=12 measurements). Source data are provided as a Source Data file.

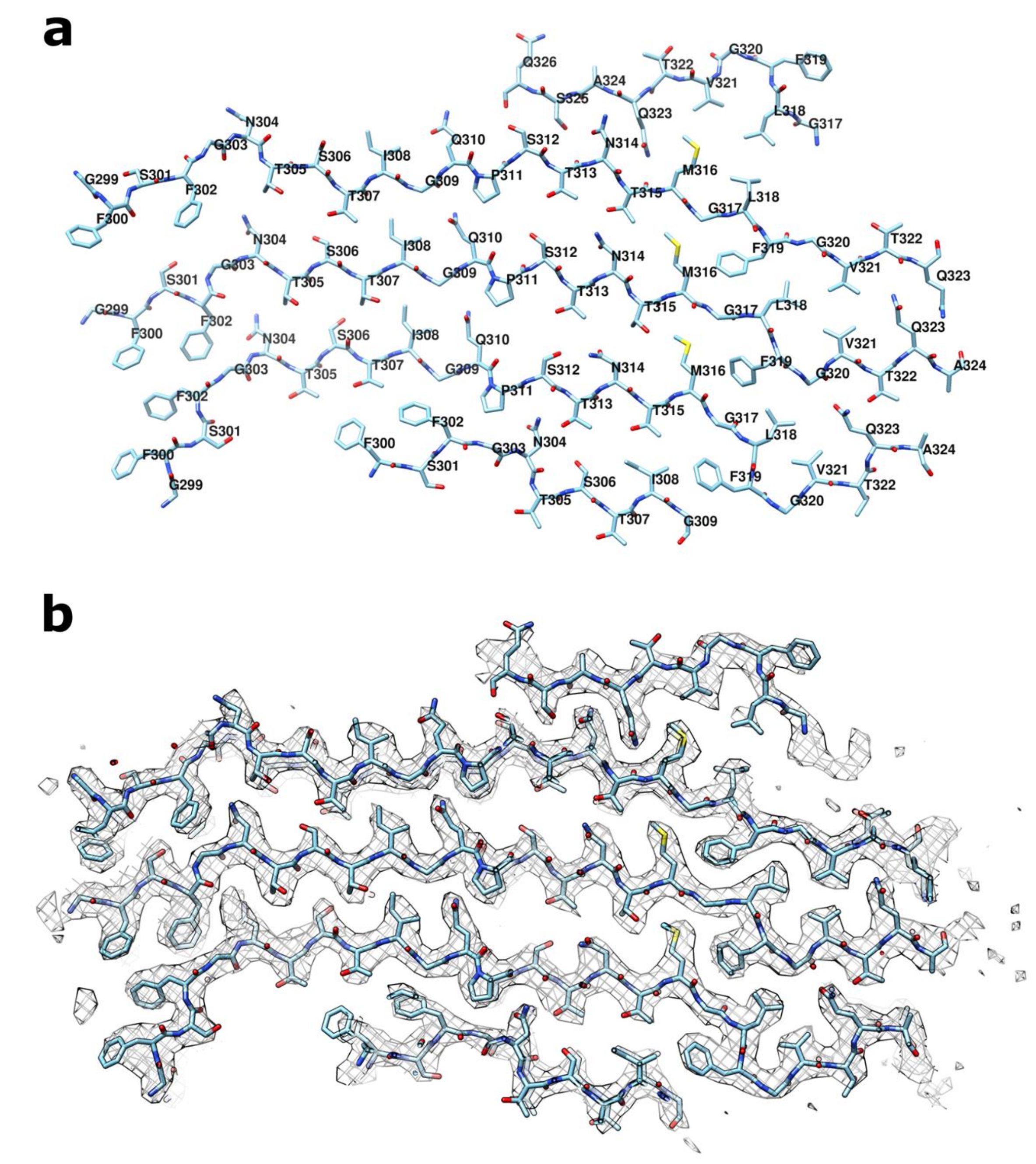

Supplementary Figure 3 | Atomic model and cryo-EM map of the Nup98^{FG}(298-327) peptide fibril. a, Single layer of the Nup98^{FG}(298-327) fibril in stick representation. Residue identifiers are shown for every amino acid. **b,** Superposition of the atomic model and the final cryo-EM map (black isomesh; contour level of 0.07).

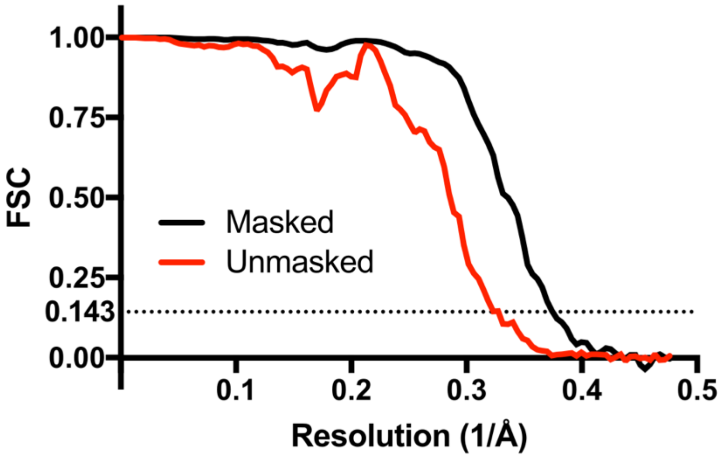

Supplementary Figure 4 | Fourier shell correlation (FSC) curves of Nup98^{FG}(298-327) peptide fibril maps. FSC curves between two independently refined masked (black) and unmasked (red) half-maps. The final resolution estimated from the value of the FSC curve for two independently refined masked half-maps at 0.143 (horizontal dashed line) is 2.67 Å. Source data are provided as a Source Data file.

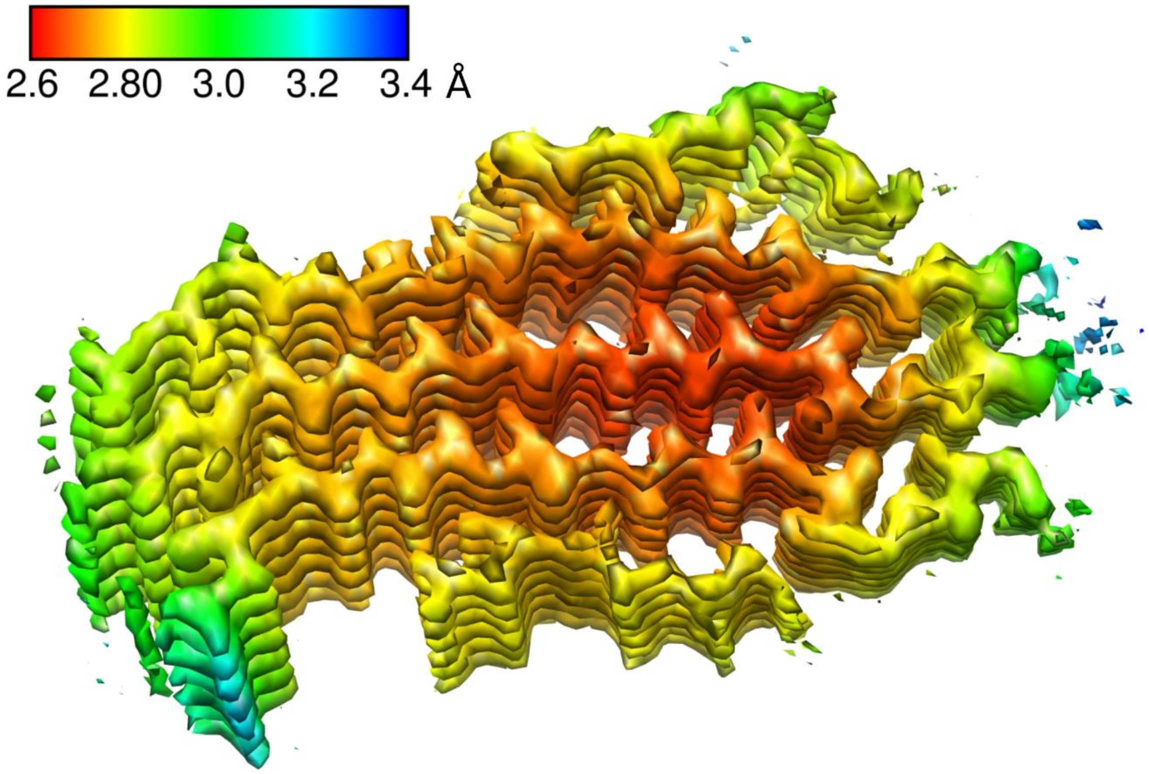

Supplementary Figure 5 | Nup98^{FG}(298-327) peptide fibril map coloured by local resolution (final global resolution: 2.67 Å).

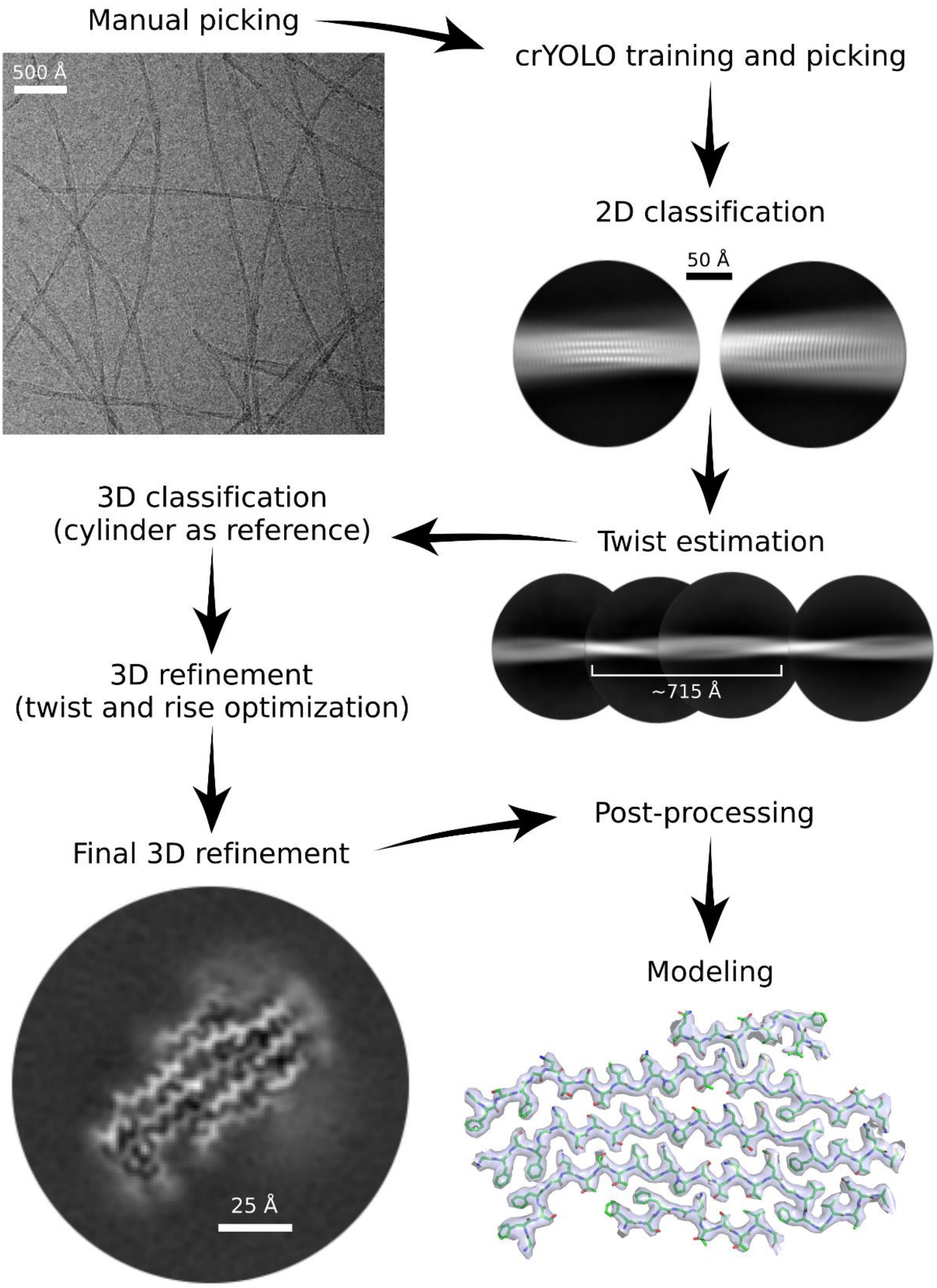

Supplementary Figure 6 | Cryo-EM data processing workflow.

Supplementary Table 1 | Cryo-EM collection, reconstruction and model building statistics.

**Data collection**		**Model building**	
Microscope	Titan Krios G2	**Model composition**	
Voltage (keV)	300	Chains	30
Detector	K3	Non-hydrogen atoms	3,450
Magnification	81,000	Protein residues	490
Pixel size (Å)	1.05	**RMS deviations**	
Defocus range (µm)	-0.7 to -2.0	Bond lengths (Å)	0.01
Exposure time (s/frame)	2.30	Bond angles (°)	1.46
Number of frames	40	**Validation**	
Total dose (e^-/Å^2)	40.62	MolProbity score	0.50
**Reconstruction**		Clashscore	0.00
Picked segments	2,877,438	Rotamer outliers (%)	0.00
Box width (pixels)	224	**Ramachandran plot**	
Inter-box distance (pixels)	18	Outliers (%)	0.00
PDB-ID	8CI8	Allowed (%)	0.00
EMDB-ID	16671	Favored (%)	100.00
Final segments	38,099		
Final resolution (Å)[a]	2.67		
Sharpening B-factor (Å^2)	-80.66		
Symmetry imposed	C1		
Helical rise (Å)	4.71		
Helical twist (°)	-1.19		

[a] The resolution was estimated from the value of the FSC curve for two independently refined half-maps at 0.143.

Supplementary Table 2 | Primers for site-directed mutagenesis.

Nup98FG F228S:
Forward: 5' CGACCGGACTGTCTGGTTCAAGTCC 3'
Reverse: 5' GGACTTGAACCAGACAGTCCGGTCG 3'

Nup98FG F319S:
Forward: 5' CAAATACCATGGGGCTGTCTGGTGTTACCCAGGCAAG 3'
Reverse: 5' CTTGCCTGGGTAACACCAGACAGCCCCATGGTATTTG 3'

Nup98FG F102S:
Forward: 5' CACCGGTACAAGCCTGTCTAGCAGCCAGAATAATG 3'
Reverse: 5' CATTATTCTGGCTGCTAGACAGGCTTGTACCGGTG 3'
